# Efficacy and survival analysis of neoadjuvant chemotherapy combined with immunotherapy in locally advanced resectable Siewert type II adenocarcinoma of the esophagogastric junction

**DOI:** 10.3389/fonc.2025.1642996

**Published:** 2025-09-16

**Authors:** Chunyue Gai, Huilai Lv, Cuili Feng, Xiaohan Zhao, Hao Wang, Bokang Sun, Fan Zhang, Ziqiang Tian

**Affiliations:** ^1^ Department of Thoracic Surgery, The Fourth Hospital of Hebei Medical University, Shijiazhuang, Hebei, China; ^2^ Hebei Key Laboratory of Accurate Diagnosis and Comprehensive Treatment of Esophageal Cancer, Shijiazhuang, Hebei, China; ^3^ Department of Endoscopy, The Fourth Hospital of Hebei Medical University, Shijiazhuang, Hebei, China; ^4^ Department of Radiation Oncology, The Fourth Hospital of Hebei Medical University, Shijiazhuang, Hebei, China

**Keywords:** Siewert type II AEG, neoadjuvant immunochemotherapy, pathological response, pathologic complete response, prognosis

## Abstract

**Background:**

Neoadjuvant chemotherapy combined with immunotherapy (nCI) has achieved significant results in esophageal and gastric cancers, but its efficacy in Siewert type II adenocarcinoma of the esophagogastric junction (AEG) remains unclear. This study aims to verify the efficacy and safety of nCI in real-world settings for locally advanced resectable Siewert type II AEG.

**Methods:**

A retrospective analysis of clinical data from 101 patients with locally advanced resectable Siewert type IIAEG who underwent esophagogastric junction resection after chemotherapy combined with Sintilimab in a single-center treatment group from December 2020 to May 2024. The analysis focused on the rates of pathological complete response (pCR), major pathological response (MPR), R0 resection rate, tumor downstaging, recurrence-free survival (RFS), and safety.

**Results:**

A total of 101 patients were included, the median follow-up time was 19.2 months. 74 patients (73.3%) experienced postoperative pathological downstaging, with 78 patients (77.2%) showing postoperative pathological T downstaging and 47 patients (55.3%) showing postoperative pathological N downstaging. Patients with cT3 had better outcomes in pCR, MPR, and postoperative pathological downstaging compared to those with cT4 (pCR 27.9% vs 12.1% p=0.044, MPR 48.8% vs 25.9% p=0.017, postoperative pathological downstaging rate 83.7% vs 65.5% p=0.041). 3–4 cycles of nCI yield a higher pathological complete response (pCR) rate compared to 1–2 cycles (26.7% vs 7.3%,*P* = 0.015).The one-year RFS rate was 93.1% (95%CI, 88.0%-98.6%), and the OS rate was 93.2% (95%CI, 88.1%~98.6%). The two-year RFS rate was 78.9% (95%CI, 69.1%-90.1%), and the OS rate was 76.0% (95%CI, 65.5%~88.2%). 4 patients (3.96%) experienced grade 3–4 TRAEs, and 7 patients (6.93%) had grade 3–4 surgical complications, with no treatment or surgery-related deaths reported.

**Conclusion:**

Preliminary results indicate that nCI shows promising efficacy in the treatment of resectable locally advanced Siewert type II AEG, with high rates of pCR and MPR, as well as good tolerance and safety. 3–4 cycles of nCI may provide better therapeutic efficacy than 1–2 cycles. These findings require confirmation in prospective head-to-head trials to establish potential long-term clinical benefits.

## Introduction

Adenocarcinoma of esophagogastric junction (AEG) refers to adenocarcinoma where the tumor center is located within 5 centimeters above or below the esophagogastric junction and crosses or contacts this line. In recent years, the epidemiological trends of AEG have shown notable changes, primarily attributed to the accelerated aging of the global population, diversified lifestyles, and complex environmental factors. According to an analysis of global gastric and esophageal cancer registry data from 2018, East Asia accounted for as much as 67.1% of new cases of AEG globally, showing a significant increase compared to data from 2012 (1, 2). The incidence of AEG in China has also shown an upward trend, consistent with the trends in Western countries, and the proportion of early cases was always less than 20% (3–5). Most patients are diagnosed at a locally advanced stage, and surgical resection remains the primary treatment for such cases. However, after surgery alone, patients often face high risks of recurrence and metastasis, leading to poor outcomes. Neoadjuvant chemotherapy (nCT) or neoadjuvant chemoradiotherapy (nCRT) can reduce tumor size, increase R0 resection rates, and extend recurrence-free survival (RFS) and overall survival (OS). These benefits have been confirmed by numerous large-scale clinical studies. nCT or nCRT combined with surgical resection is gradually becoming the standard treatment model for locally advanced AEG.

In clinical practice, the combination of programmed cell death ligand 1 (PD-L1) inhibitors and chemotherapy has achieved significant results in the treatment and long-term prognosis of patients with advanced upper gastrointestinal tumors, including AEG (6–8). This approach provides a new first-line treatment option for patients with advanced AEG. The oncological efficacy advantages obtained in advanced upper gastrointestinal tumors have also sparked enthusiasm for exploring new adjuvant immunotherapy regimens. Some prospective phase II and phase III clinical trials have confirmed that preoperative nCI shows notable benefits in terms of safety and improved pathological response rates in gastric or esophageal cancer (9–12).

Lately, the impressive findings from the KEYNOTE-062 and CheckMate-649 clinical trials (13, 14) have confirmed the therapeutic efficacy of immunotherapy in combination with chemotherapy for unresectable advanced gastric/gastroesophageal junction(G/GEJ) cancer.These positive outcomes provide a solid rationale for exploring the application of this treatment strategy in the neoadjuvant therapy setting for patients with advanced G/GEJ cancer. While,Siewert type II AEG is demarcated by a virtual anatomical boundary within the esophagogastric junction region, and its unique anatomical location particularity entails a host of complexities and challenges,with prognosis influenced by tumor stage, nodal status, and resection margins (15). There is considerable controversy regarding its treatment strategies, including the pattern of lymph node metastasis, surgical approach, and extent of resection. Besides, the efficacy of nCI in Siewert type II AEG has not yet been established and lack of large-sample data support. This study aims to verify the efficacy and safety of nCI in real-world settings for locally advanced resectable Siewert type II AEG, and to further analyze the factors related to efficacy and safety.

## Methods

### Study design and patients

This study screened patients with locally advanced resectable Siewert type II AEG who were registered in the thoracic surgery single-treatment group database of the Fourth Hospital of Hebei Medical University from December 2020 to May 2024 and received nCI. Inclusion criteria were: (1) adult patients (age ≥18 years); (2) histologically confirmed adenocarcinoma with clinically stage II-III Siewert type II AEG, (3) expected to be surgically resectable; (4) completed nCI for 1–4 cycles. Exclusion criteria: (1) clinical stage T4b, (2) Incomplete medical record. Ethical approval was obtained from the Ethics Committee of the Fourth Hospital of Hebei Medical University and conducted in accordance with the practice of the Declaration of Helsinki. Informed consent from patients was waived because of the retrospective nature of this study, which was approved by the Ethics Committee of the Fourth Hospital of Hebei Medical University.

### Staging

Clinical staging was performed by chest and abdominal CT enhancement scans, endoscopic ultrasound (EUS), and neck lymph node ultrasound at baseline, every two cycles neoadjuvant and before surgical resection, with PET/CT examination when necessary. Clinical and pathological staging was determined according to the AJCC 8th edition gastric cancer TNM staging system.

### Treatment and follow-up

All patients completed the SOX scheme (S-1, 40mg/m2 orally twice daily for 14 days followed by 7 days off; Oxaliplatin, 130mg/m2 intravenously on day 1 every 3 weeks) in combination with the Sintilimab Injection(200mg intravenous infusion over 30 minutes on Day 1 of each chemotherapy cycle), followed by efficacy evaluation. Surgery was performed 3–4 weeks after the last dose of nCI to avoid overlapping chemotherapy or immunotherapy-related toxicity with perioperative complications. Surgical options include laparoscopic combined with left/right thoracotomy and laparotomy esophagogastric junction cancer resection. Routine follow-up is conducted every three months within the first year post-surgery, every six months from years one to three, and annually after three years. The final follow-up was completed on October 31, 2024.

### Observation indicators

The primary endpoints were pathological complete response (pCR) and major pathological response (MPR), while the secondary endpoints were recurrence-free survival (RFS),overall survival (OS),and treatment-related adverse reactions (TRAEs). pCR is defined as the absence of residual tumor cells in all resected tumor specimens and regional lymph nodes after completing neoadjuvant therapy and surgical resection. MPR is defined as having less than 10% residual tumor cells within the primary tumor bed after neoadjuvant therapy and surgical resection. Pathology was assessed by two experienced gastrointestinal pathologists. Discrepancies in assessment were resolved through a consensus conference involving a third senior pathologist.OS is defined as the time from the date of surgery until the patients’ death for any reason. RFS is defined as the time from the date of surgery until recurrence or the end of follow-up (non-disease-related deaths are considered censored). R0 resection is defined as a microscopic negative margin resection in which there are no visible or microscopic tumors in the primary tumor bed. Postoperative pathological staging was compared with baseline clinical staging, and a decrease in T stage, N stage, or both is defined as tumor downstaging. Safety assessment was based on the National Cancer Institute Common Toxicity Criteria (NCI-CTCAE, 5.0 version). Additionally, Additionally, surgical complications were classified was based on the Clavien-Dindo scale (2009 version).

### Statistical analysis

Statistical analysis was conducted after data collection and validation were completed. The complete demographic information and baseline characteristics of the patient population were compiled into tables and analyzed. Categorical variables were expressed as numbers and percentages. Statistical analysis was performed on the efficacy and safety of treatment for all patients, with results presented in the form of numbers and percentages. Comparisons between subgroups were conducted using the Chi-square test or Fisher’s exact test. The median follow-up time was calculated using the reverse Kaplan-Meier method. RFS and OS were analyzed using the Kaplan-Meier method. Assessing the Impact of tumor-related death on cancer-specific survival using a Competing Risks Model and comparing intergroup differences via the Gray Test.All statistical tests were two-tailed, and P<0.05 was considered statistically significant. All statistical analyses were conducted using SPSS (version 26.0; SPSS Inc., Chicago, IL, USA) and R software(version 4.3.1).

## Results

### Baseline characteristics

A total of 101 patients with locally advanced Siewert type II AEG who underwent surgical treatment at this center from December 2020 to May 2024 met the inclusion and exclusion criteria for this study. Among them, there were 89 males (88.1%) and 12 females (11.9%), with a median age of 65 years (IQR 59.5-70.0 years). The cTNM stage was Phase II B in 16 cases (15.8%), and PhaseIIIin 85 cases (84.2%). All cT stages were cT3 or cT4, with 43 cases (42.6%) at cT3 and 58 cases (57.4%) at cT4. Of the cases, 16 were classified as cN0 (15.5%), 58 as cN1 (57.4%), 25 as cN2 (24.8%), and 2 as cN3 (2.0%). (**Table 1**).

**Table 1:** Baseline characteristics.

Characteristics	No. (%)
Age (years)	
Median (IQR)	65 (59.5-70.0)
<65	45 (44.6%)
≥65	56 (55.4%)
Sex	
Female	12 (11.9%)
Male	89 (88.1%)
Smoking	
Yes	47 (46.5%)
No	54 (53.5%)
Alcohol Drinking	
Yes	25 (24.8%)
No	76 (75.2%)
Clinical TNM Stage	
II B	16 (15.8%)
III	85 (84.2%)
Clinical T Stage	
T3	43 (42.6%)
T4	58 (57.4%)
Clinical N Stage	
N0	16 (15.8%)
N1	58 (57.4%)
N2	25 (24.8%)
N3	2 (2.0%)
nCI Cycle Numbers*	
1–2 cycles	41 (40.6%)
3–4 cycles	60 (59.4%)

*nCI: neoadjuvant chemotherapy combined with immunotherapy.

### Neoadjuvant therapy and results

A total of 41 patients (40.6%) received 1–2 cycles of neoadjuvant therapy, while 60 patients (59.4%) received 3–4 cycles. Treatment cycle primarily depends on the surgeon’s decision and tolerance to neoadjuvant treatment. Postoperative pathological analysis showed that 19 patients (18.8%) achieved pCR (ypT0N0), 2 patients (1.98%) were ypT0N+ responders, and 36 patients (35.6%) achieved MPR. Additionally, 74 patients (73.3%) achieved pathological downstaging postoperatively, including 78 patients (77.2%) with T-staging downstaged and 47 patients (55.3%) with N-staging downstaged (**Table 2**).

**Table 2:** Postoperative pathological analysis.

Pathological status	No.(%)
nCI	
Pathologic Complete Response (pCR)	19(18.8%,95%CI,12.1%fendo~27.3%)
Major Pathologic Response (MPR)	36(35.6%,95%CI,26.8%fendo~45.3%)
Pathologic Downstaging	74(73.3%,95%CI,64.1%fendo~81.2%)
Pathologic Downstaging in T category	78(77.2%,95%CI,68.4%fendo~84.6%)
Pathologic Downstaging in N category*	47(55.3%,95%CI,44.7%fendo~65.5%)

*85 cases were N+ before treatment.

### Surgical treatment

In all 101 patients, surgery was performed as planned, with 100 cases (99%) successfully achieving R0 resection. Only one patient failed to meet the R0 resection criteria because enlarged lymph nodes found behind the pancreas during surgery prevented complete resection, and postoperative pathological examination of two excised enlarged lymph nodes revealed lymph node metastasis. The median interval between the end of neoadjuvant therapy and surgery was 40 days (IQR 35–51 days). 91 cases (90.1%) underwent combined upper abdominal and left thoracotomy approach, 8 cases (7.9%) underwent open abdominal surgery, and 2 cases (2.0%) underwent combined upper abdominal and right thoracotomy approach. The median surgical time was 266 minutes (IQR 235.0-300.5min), and the average intraoperative blood loss was 150ml (IQR 100–200 ml). The median hospital stay was 9 days (IQR 8.5-11.0 days), and the median number of lymph nodes removed was 28 (IQR 19-40.5).In terms of surgical approach, 91 cases (90.1%) underwent upper abdominal combined with left thoracotomy surgery, 8 cases (7.9%) underwent open abdominal surgery, and 2 cases (2.0%) underwent upper abdominal combined with right thoracotomy surgery (**Table 3**).

**Table 3:** Surgical treatment details of Siewert type II AEG patients receiving neoadjuvant chemotherapy and immunotherapy.

Analytical indicators	Results
Surgical time (min)	266 (IQR 235.0-300.5)
Intraoperative blood loss (ml)	150 (IQR 100-200)
New adjuvant-surgery interval (d)	40 (IQR 35-51)
Number of days in hospital (d)	9 (IQR 8.5-11.0)
Number of lymph node dissections (count)	28 (IQR 19-40.5)
Surgical approach	
Upper abdominal + Left Thoracotomy	91 (90.1%)
Upper abdominal + Right Thoracotomy	2 (2.0%)
Upper abdominal	8 (7.9%)

### Exploratory analysis for factors influence efficacy of nCI patients

This study further explored various factors that may influence the efficacy of surgery for nCI patients, as detailed in **Table 4**. The results showend that patients with cT3 had better outcomes in pCR, MPR, and postoperative pathological downstaging compared to those with cT4 (pCR:27.9%[95%CI,16.3-42.4] vs 12.1%[95%CI,5.6-22.2], p=0.044, MPR:48.8% [95%CI,34.4-63.4] vs 25.9%[95%CI,16.0-38.1],p=0.017, postoperative pathological downstaging rate 83.7%[95%CI,70.7-92.4] vs 65.5%[95%CI,52.8-76.8],p=0.041). Additionally, patients who completed 3–4 cycles of nCI had a better pCR rate than those who completed 1–2 cycles (26.7% [95%CI,16.8-38.8] vs 7.3% [95%CI,2.1-18.3],p=0.015).

**Table 4:** The factors of affecting the efficacy of neoadjuvant chemotherapy and immunotherapy for Siewert type II AEG patients.

Factors	Subgroup	pCR, n (% [95% CI])	P-value	MPR, n (% [95% CI])	P-value	Postoperative Pathologic Downstaging, n (% [95% CI])	P-value
Age	<65 (n=45)	10 (22.2 [12.0-35.8])	0.432	17 (37.8 [24.7-52.3])	0.688	34 (75.6 [61.7-86.3])	0.641
	≥65 (n=56)	9 (16.1 [8.3-27.3])		19 (33.9 [22.6-46.9])		40 (71.4 [58.7-82.0])	
Sex	Female (n=12)	2 (16.7 [3.6-43.6])	1.000	2 (16.7 [3.6-43.6])	0.254	11 (91.7 [67.2-99.1])	0.235
	Male (n=89)	17 (19.1 [12.0-28.2])		34 (38.2 [28.6-48.5])		63 (70.8 [60.8-79.5])	
Smoking	Yes (n=47)	8 (17.0 [8.4-29.6])	0.667	19 (40.4 [27.3-54.7])	0.349	32 (68.1 [54.0-80.0])	0.272
	No (n=54)	11 (20.4 [11.3-32.5])		17 (31.5 [20.3-44.6])		42 (77.8 [65.4-87.2])	
Alcohol Drinking	Yes (n=25)	4 (16.0 [5.7-33.7])	0.905	12 (48.0 [29.5-66.9])	0.137	19 (76.0 [57.1-89.3])	0.722
	No (n=76)	15 (19.7 [12.0-29.7])		24 (31.6 [22.0-42.6])		55 (72.4 [61.6-81.5])	
Clinical Stage	II B (n=16)	3 (18.8 [5.6-42.1])	1.000	6 (37.5 [17.4-61.7])	0.866	10 (62.5 [38.3-82.6])	0.451
	III (n=85)	16 (18.8 [11.6-28.1])		30 (35.3 [25.8-45.8])		64 (75.3 [65.4-83.5)	
Clinical T stage	T3 (n=43)	12 (27.9 [16.3-42.4])	**0.044**	21 (48.8 [34.4-63.4])	**0.017**	36 (83.7 [70.7-92.4])	**0.041**
	T4 (n=58)	7 (12.1 [5.6-22.2])		15 (25.9 [16.0-38.1])		38 (65.5 [52.8-76.8])	
Clinical N stage	N0 (n=16)	3 (18.8 [95-42.1])	1.000	6 (37.5 [17.4-61.7])	0.629	10 (62.5 [38.3-82.6])	0.163
	N1 (n=58)	11 (19.0 [10.5-30.4])		23 (39.7 [27.8-52.5])		47 (81.0 [69.6-89.5])	
	N2 (n=25)	5 (20.0 [8.1-38.4])		7 (28.0 [13.5-47.3])		16 (64.0[44.5-80.5])	
	N3 (n=2)	0 (0)		0 (0)		1 (50.0 [6.4-93.9])	
Cycle Numbers	1-2 (n=41)	3 (7.3 [2.1-18.3])	**0.015**	16 (39.0 [25.3-54.3])	0.558	28 (68.3 [53.2-80.9])	0.350
	3-4 (n=60)	16 (26.7 16.8-38.8])		20 (33.3 [22.4-45.8])		46 (76.7 [64.9-86.0])	
PD-L1 Expression	CPS≥10 (n=6)	0	1	0	0.298	3 (50.0[16.7%-83.3])	0.644
	CPS<10 (n=20)	2 (10.0[2.1%-28.4%])		5 (25.0[10.2%-46.4])		13 (65.0[43.2%-82.8])	

Bold values indicates with statistic significant.

### Follow-up

**Figure 1:**
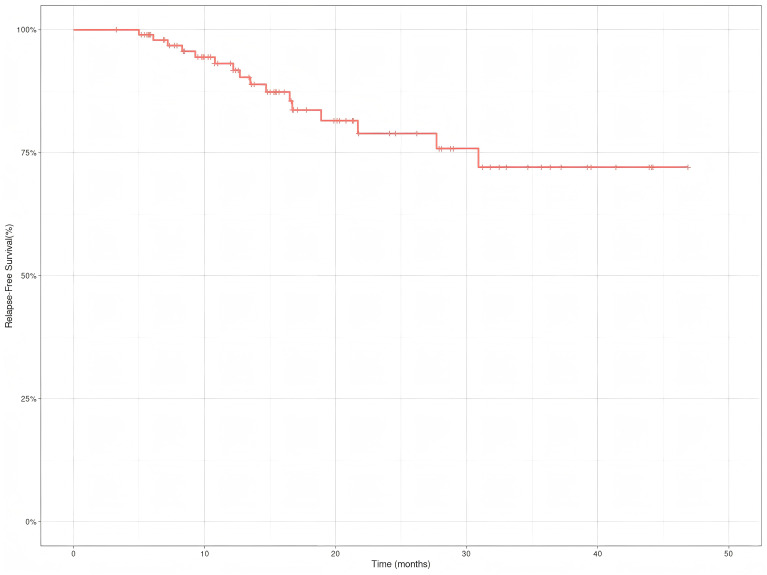
Relapse-free survival(RFS) of Siewert type II adenocarcinoma of the esophagogastric junction patients receiving neoadjuvant chemotherapy combined with immunotherapy.

**Figure 2:**
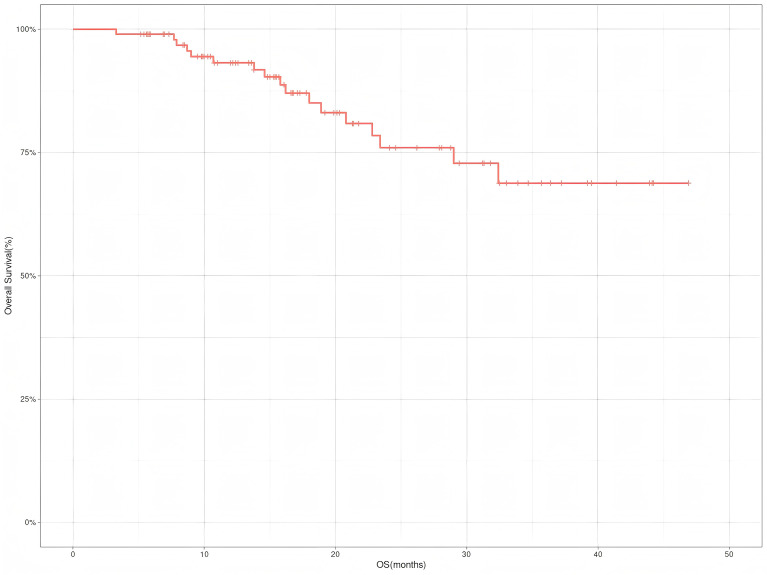
Overall survival (OS) of Siewert type II adenocarcinoma of the esophagogastric junction patients receiving neoadjuvant chemotherapy combined with immunotherapy.

Interim analysis results indicate:Data as of October 31,2024, showed a median follow-up duration of 19.2 months (IQR:12.4-31.8 months). The one-year RFS rate was 93.1% (95%CI, 88.0%-98.6%), and the OS rate was 93.2% (95%CI, 88.1%~98.6%). The two-year RFS rate was 78.9% (95%CI, 69.1%-90.1%), and the OS rate was 76.0% (95%CI, 65.5%~88.2%) (**Figures 1**, **2**). Neither the median RFS or OS reached the target endpoint.

By the final follow-up, a total of 17 patients had died, including 11 deaths due to recurrence or metastasis, and 6 patients were non-disease-related deaths:3 patients died from cardiovascular diseases, 1 patient died from COVID-19, and 2 patients died from severe pneumonia. Additionally, 5 patients have experienced recurrence or metastasis and are still under follow-up treatment. Among these 16 patients with metastasis or recurrence, all had a baseline clinical stage of cT4aN+, and all achieved R0 resection but did not achieve pCR. The most common patterns of recurrence were anastomotic recurrence (6 cases, 37.5%), abdominal lymph node recurrence (4 cases, 25.0%), bone metastasis (2 cases, 12.5%), liver metastasis (2 cases, 12.5%), chest/abdominal wall muscle metastasis (2 cases, 12.5%), and lung metastasis (1 case, 6.25%). In the pCR cases, there were no recurrences or deaths,and in the non-pCR cases, 16 patients(19.51%)had recurrence or metastasis, 11 patients(13.41%) died from tumor-related causes. Among the MPR cases, 2 patients had recurrence or metastasis, no patient died from tumor-related causes, and the non-MPR cases, 14 patients (21.5%)had recurrence or metastasis, and 11 patients(16.9%) died from tumor-related causes.The prognosis of the MPR group is better than that of the non-MPR group, and the difference is statistically significant(Recurrence or metastasis 5.56% vs 21.5% p=0.035, Disease-related death 0% vs 16.9% p=0.023) (**Table 5** **).**

**Table 5:** Recurrence and metastasis situation for Siewert type II AEG patients receiving neoadjuvant chemotherapy and immunotherapy.

	Recurrence or metastasis	P-value	Disease-related death	P-value
pCR	0	0.080	0	0.200
non-pCR	16 (19.5%)		11 (13.4%)	
MPR	2 (5.6%)	**0.035**	0	**0.023**
non-MPR	14(21.5%)		11 (16.9%)	

Bold values indicates with statistic significant.

**Figure 3:**
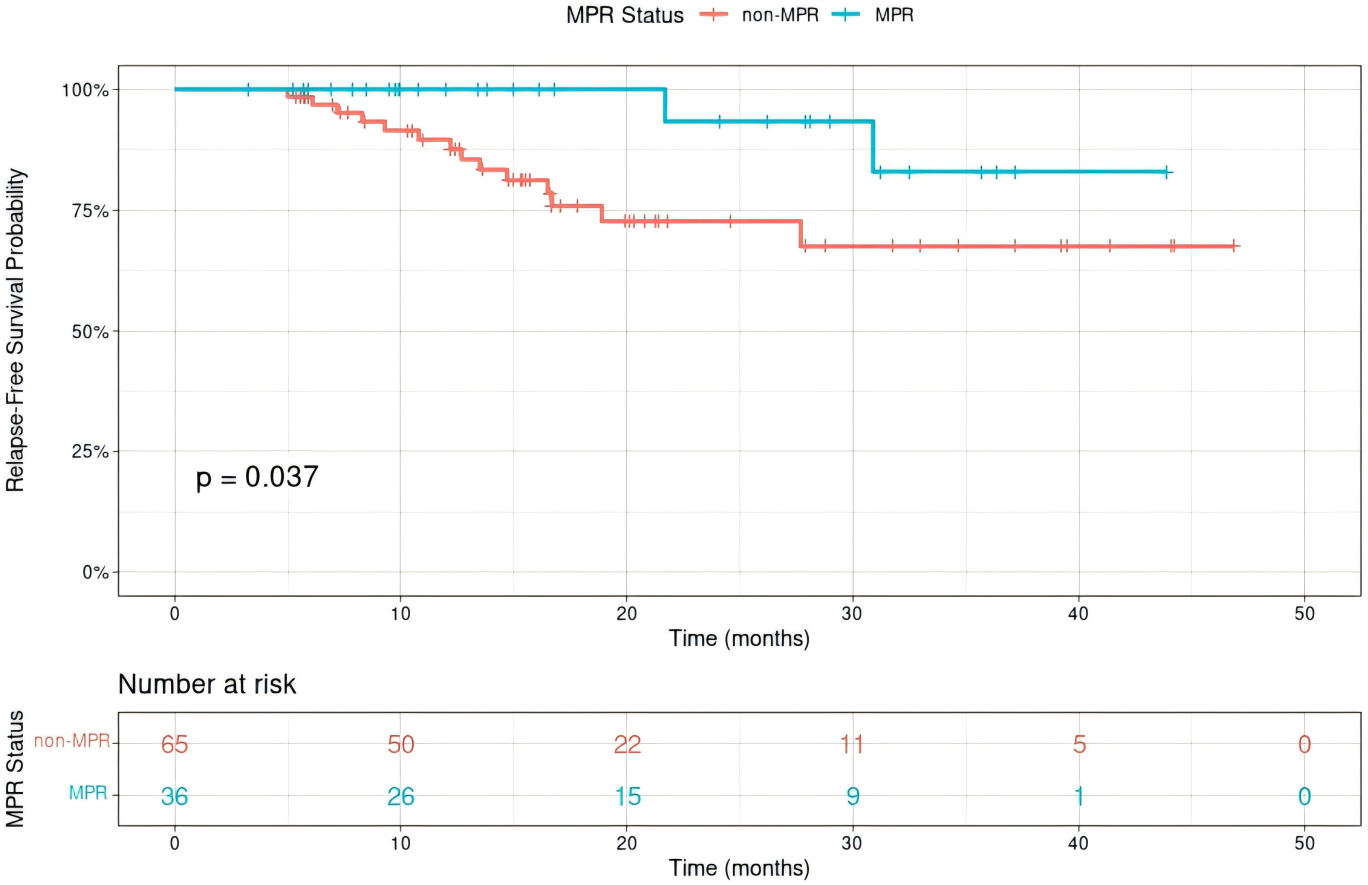
Relapse-free survival(RFS) of Siewert type II adenocarcinoma of the esophagogastric junction patients receiving neoadjuvant chemotherapy combined with immunotherapy for those with a major pathological response (MPR) and non-MPR.

**Figure 4:**
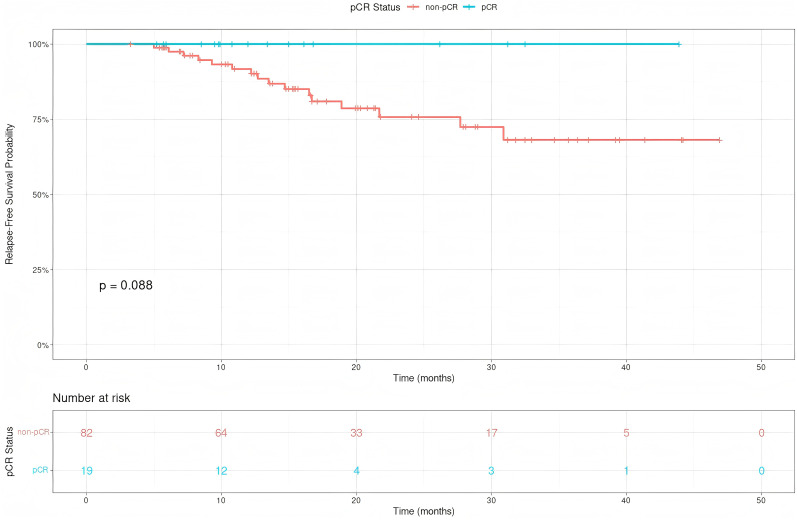
Relapse-free survival(RFS) of Siewert type II adenocarcinoma of the esophagogastric junction patients receiving neoadjuvant chemotherapy combined with immunotherapy for those with a pathological complete response(pCR) and non-pCR.

Median RFS was not reached in MPR or non-MPR groups. MPR group patients had significantly better RFS than non-MPR group (p=0.037, HR = 0.23, 95% CI 0.05-1.04**;** **Figure 3** **).** For pCR vs. non-pCR groups, median RFS was not reached, with no significant RFS difference (p=0.088, HR = 0.17, 95% CI 0.01-3.04; **Figure 4** **).**

**Figure 5:**
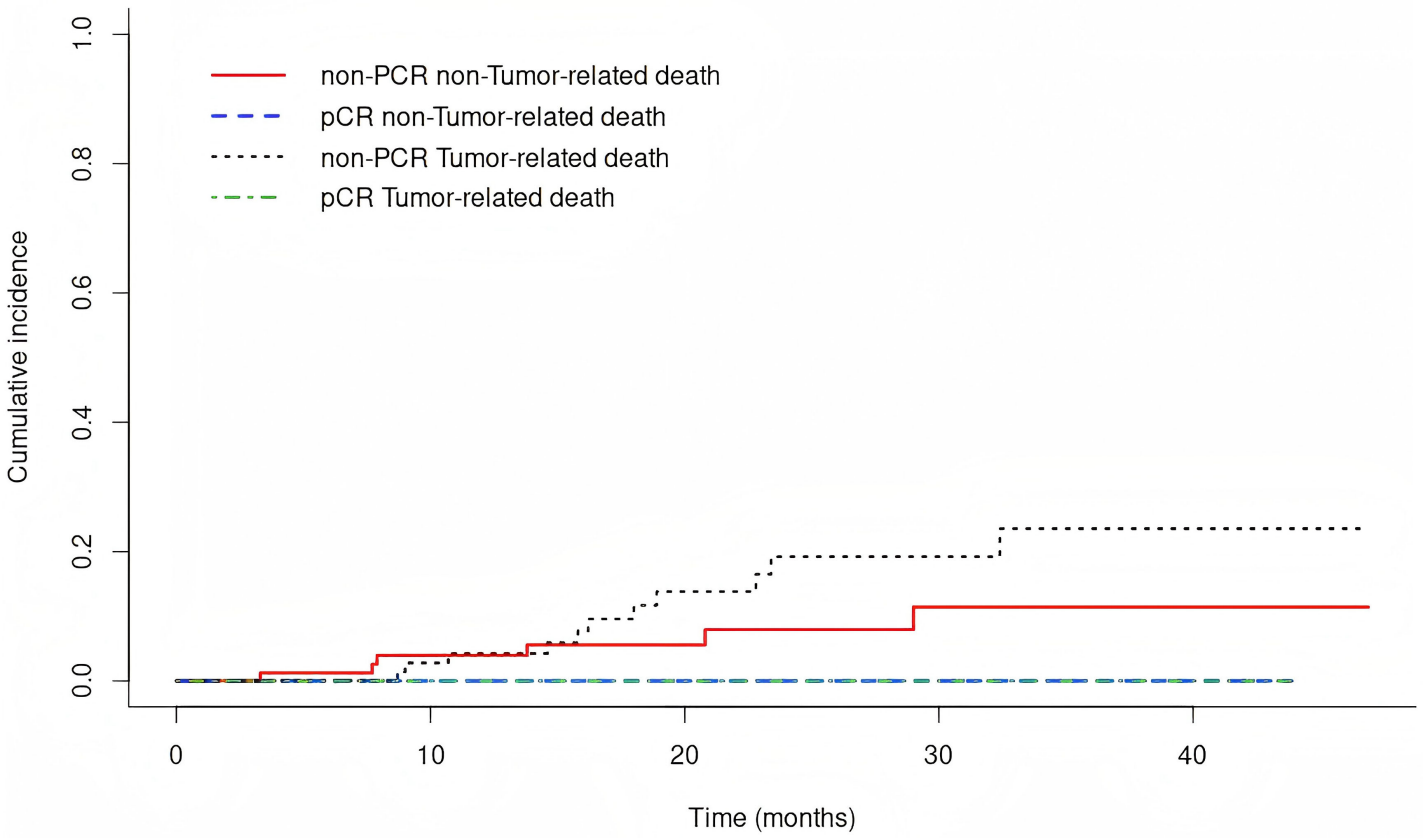
The cumulative incidence of tumor-related death Siewert type II adenocarcinoma of the esophagogastric junction patients receiving neoadjuvant chemotherapy combined with immunotherapy for those with a pCR and non-pCR.

**Figure 6:**
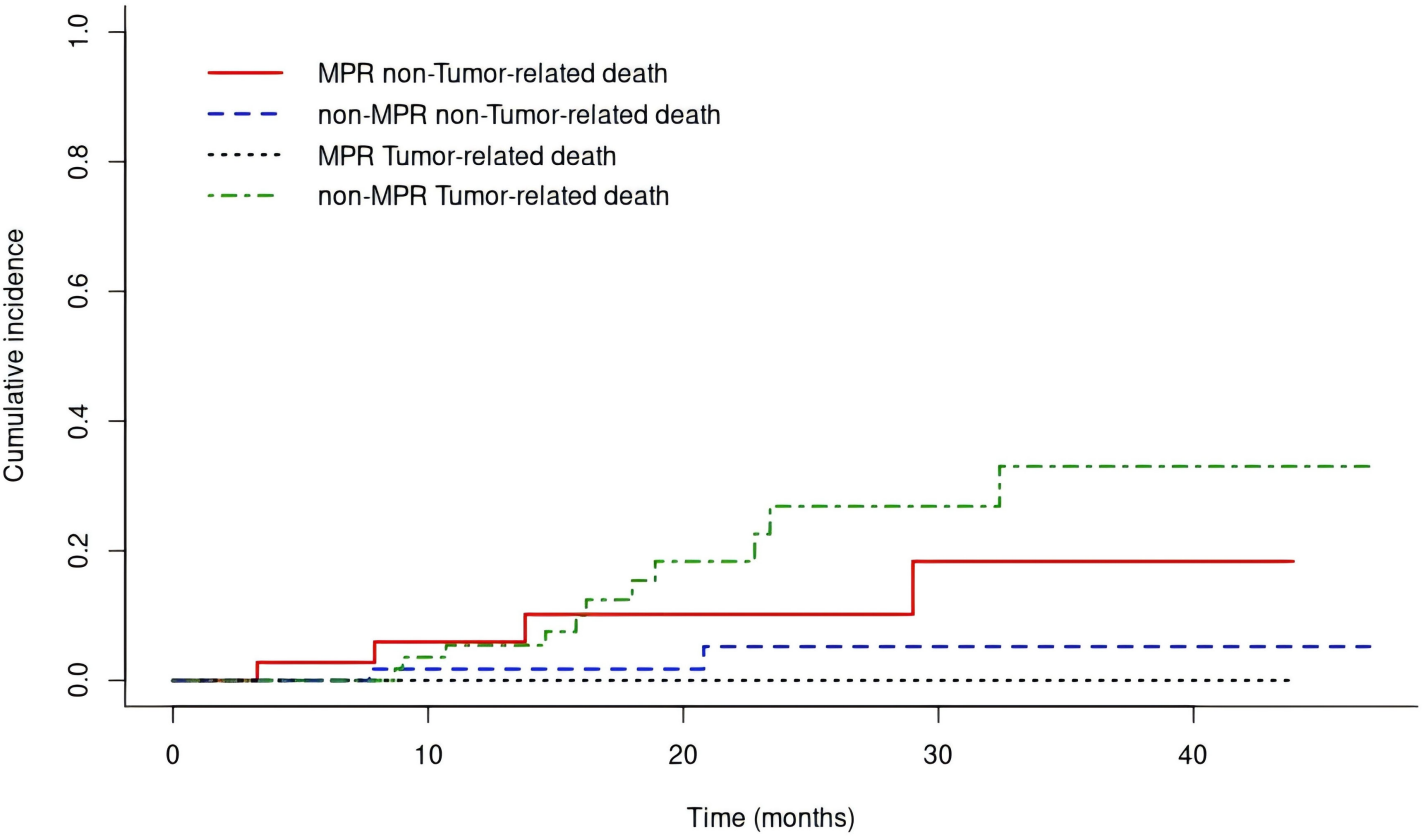
The cumulative incidence of tumor-related death Siewert type II adenocarcinoma of the esophagogastric junction patients receiving neoadjuvant chemotherapy combined with immunotherapy for those with a MPR and non-MPR.

Tumor-related death cumulative incidence: in non-pCR group, 1-year 4.2% (95% CI 0.0-9.0%), 2-year 19.2% (95% CI 7.9-30.5%); in pCR group, both rates 0%. Difference not significant (Gray’s test P = 0.199; **Figure 5**). In non-MPR group, 1-year 5.4% (95% CI 0.0-11.5%), 2-year 26.8% (95% CI 11.1-42.6%); in MPR group, both rates 0%. Difference significant (Gray’s test P = 0.008**;** **Figure 6** **).**

### Treatment safety

A total of 36 patients experienced TRAEs, among which 16 cases involved leukopenia, 17 cases involved nausea or vomiting, 2 patients involved diarrhea, and 1 patient had drug-induced liver injury. The occurrence of immune-related adverse reactions (irAEs) is as follows: 3 cases of immune-related hypothyroidism, 2 cases of immune-related hyperthyroidism, 1 cases of Immune myocarditis and 1 case of Immune myositis with myasthenia gravis. The myocarditis case developed three weeks after the first cycle of immunotherapy, presenting with elevated troponin and arrhythmia. The patient responded well to high-dose glucocorticoids and recovered without long-term cardiac dysfunction, allowing surgery four weeks later. The case of immune myositis initially presented with blepharoptosis progressing to limb weakness, accompanied by elevated creatine kinase levels and confirmed by muscle biopsy. Treatment with glucocorticoids and cholinesterase inhibitors led to significant improvement, enabling successful surgery.

The incidence rate of grade 3–4 adverse reactions was 4.95% and no treatment-related death occurred. (**Table 6**). Besides, clinicopathological factors such as the number of chemotherapy cycles, age, gender, and TNM stage showed no significant influence on the occurrence of grade 3–4 adverse reactions (all P > 0.05).

**Table 6:** Adverse events for Siewert type II AEG patients receiving neoadjuvant chemotherapy and immunotherapy.

Adverse events	Adverse event grading		Numbers of nCI cycles		
	Grade 1-2	Grade 3-4	1-2Cycles (n=41)	3-4Cycles (n=60)	P-value
Any treatment-related adverse event	31 (30.69%)	5 (4.95%)	16 (39.02%)	20 (33.33%)	0.56
Immunologically associated thyroid dysfunction	4 (3.96%)	1 (0.99%)	2 (4.88%)	3 (5%)	1
Immune myositis	0	1 (0.99%)	1 (2.44%)	0	0.41
Immune myocarditis	0	1 (0.99%)	1 (2.44%)	0	0.41
Abnormal liver function	1 (0.99%)	0	1 (2.44%)	0	0.41
White blood cell count decreased	15 (14.85%)	1 (0.99%)	6 (14.63%)	10 (16.67%)	0.78
Diarrhoea	1 (0.99%)	1 (0.99%)	1 (2.44%)	1 (1.67%)	1
Nausea, vomiting	17 (16.83%)	0	7 (17.07%)	10 (16.67%)	0.96

nCI, neoadjuvant chemotherapy combined with immunotherapy.

### Surgical complications

Postoperative complications occurred in 37 patients. 4 patients experienced postoperative respiratory failure, 2 patients developed arrhythmias, and 4 patients had anastomotic stricture. 1 patient experienced anastomotic bleeding. A total of 29 patients developed postoperative pneumonia, with 3 cases requiring endotracheal intubation with ventilator-assisted breathing. 5 patients developed postoperative deep vein thrombosis in the lower extremities. No intraoperative complications or deaths related to postoperative complications were reported (**Table 7**). In addition, the number of treatment cycles was also associated with increased postoperative complication rates.

**Table 7:** Postoperative complications.

	Adverse event grading		Numbers of nCI cycles		
	Grade 1-2	Grade 3-4	1-2Cycles (n=41)	3-4Cycles (n=60)	P-value
Any Complications	33 (32.67%)	4 (3.96%)	14 (34.15%)	23 (38.33%)	0.67
Failure of respiration	1 (0.99%)	3 (2.97%)	2 (4.88%)	2 (3.33%)	1
Arhythmia	2 (1.98%)	0	1 (2.44%)	1 (1.67%)	1
Stenosis of the anastomosis	4 (3.96%)	2 (1.98%)	4 (9.76%)	2 (3.33%)	0.36
Anastomotic bleeding	1 (0.99%)	0	0	1 (1.67%)	1
Pulmonary infection	26 (25.74%)	3 (2.97%)	12 (29.27%)	17 (28.33%)	0.92
Deep vein thrombosis of the lower extremities	5 (4.95%)	0	2 (4.88%)	3 (5%)	1

nCI, neoadjuvant chemotherapy combined with immunotherapy.

## Discussion

At present, the standard neoadjuvant treatment for locally advanced Siewert type II AEG remains based on chemotherapy or chemoradiotherapy with fluorouracil combined with platinum agents. The MAGIC study (16)demonstrated that perioperative ECF chemotherapy improved overall survival compared with surgery alone, with a 5-year OS of 36% versus 23%. The EORTC 40954 trial (17) confirmed that neoadjuvant FLP chemotherapy improved R0 resection rates (81.9% vs. 66.7%) but did not show a survival benefit. More recently, the FLOT4-AIO trial (18)established the FLOT regimen as a standard perioperative option, with a pCR rate of 16% and an R0 resection rate of 85%。

Specifically for Siewert II/III AEG, retrospective analyses have shown even lower pathological responses with nCT alone, with pCR rates around 3% and R0 resection rates of 82%, leading to inferior survival compared with nCRT (3-year OS 58.0% vs. 79.2%) (17, 19). Although nCRT can improve tumor regression, it is often associated with severe toxicities that compromise tolerance and compliance. As a result, many patients fail to complete the planned regimen, potentially missing the opportunity for curative surgery. Therefore, more effective and tolerable treatment strategies are urgently needed to enhance efficacy and prognosis in locally advanced Siewert type II AEG.

In recent years, immunotherapy has brought new breakthroughs, several studies such as ORIENT-16, ATTRACTION-4, CheckMate-649 (8, 13, 20) have indicated that the combination of immunotherapy with chemotherapy can improve the efficacy and prognosis of advanced gastric cancer, making it a new option for the treatment of locally advanced AEG. Several small-sample phase II clinical trials (12, 21), such as NEOSUMMIT-01 and NEOSUMMIT-03, have demonstrated that neoadjuvant chemotherapy combined with immunotherapy shows promising application prospects in terms of pCR rate, MPR rate, and safety for locally advanced gastric cancer or esophagogastric junction cancer. However, most of these studies involved small sample sizes and did not exclusively enroll Siewert type II AEG patients,the value of nCI in locally advanced Siewert type II AEG remains unclear. Therefore, we conducted this study to evaluate the feasibility and safety of a new neoadjuvant treatment regimen combining chemotherapy with ICIs for patients with resectable locally advanced Siewert type II AEG. We observed a pCR rate of 18.8%, an MPR rate of 35.6%, and an R0 resection rate of 94.1%. Compared with historical nCT trials, these results suggest at least comparable, and potentially superior, pathological outcomes, although cross-trial comparisons should be interpreted with caution.

This study demonstrates that the neoadjuvant treatment regimen combining fluorouracil/tegafur with platinum-based chemotherapy and ICIs shows significant efficacy for resectable locally advanced Siewert type II AEG. Overall, the pCR rate was 18.8%, the MPR rate was 35.6%, and the pathological downstaging rate was 73.3%. Additionally, among clinical T stages, patients with cT3 stage showed better outcomes compared to those with cT4 stage in terms of pCR, MPR, and postoperative pathological downstaging (pCR 27.9% vs 12.1%, p=0.044; MPR 48.8% vs 25.9%, p=0.017; postoperative pathological downstaging rate 83.7% vs 65.5%, p=0.041). At present, the assessment of tumor regression response is of notable importance for determining treatment efficacy, formulating subsequent treatment plans, and predicting patient prognosis (22). It also serves as a good predictor for recurrence, metastasis, and survival following neoadjuvant therapy for tumors (23–25). The pCR and MPR rates observed in this study (18.8% and 35.6%, respectively) were higher than those observed in some previous clinical studies of nCT and neoadjuvant monotherapy immunotherapy, which ranged from 4.0% to 6.3% (26–29) and 16.1% to 30% (18, 29). This suggests that the nCI may be more effective than chemotherapy or immunotherapy alone. This may be attributed to the regulatory effect of chemotherapy drugs on the immune status of tumor microenvironment, thereby promoting the release of tumor antigens (30–32). The synergistic effect of immunotherapy and chemotherapy has likely contributed to the higher pCR rates and MPR rates.

With a median follow-up of 19.2 months, the median RFS and OS had not yet been reached, underscoring the relatively immature survival data at this stage. Accordingly, the present results should be regarded as an interim analysis. Patients in the MPR group experienced significantly better RFS compared with the non-MPR group (p=0.037). In contrast, although the pCR group showed a numerical trend toward improved RFS (p=0.088), this difference did not reach statistical significance. The cumulative incidence of tumor-related death further highlighted this pattern: in the non-MPR group, it was 5.4% (95% CI: 0.0–11.5%) at 1 year and 26.8% (95% CI: 11.1–42.6%) at 2 years, whereas no tumor-related deaths occurred in the MPR group at either timepoint (Gray’s test, p=0.008). However, no significant difference was observed between the pCR and non-pCR groups. Several factors may account for this discrepancy. First, the relatively small sample size reduces the statistical power to detect survival differences, particularly when stratifying by pathological response. Second, the short follow-up period, with median RFS not yet reached, results in immature survival data. Third, non-cancer-related deaths and the impact of postoperative adjuvant therapy may have diluted the prognostic effect of pCR. Nevertheless, the observed association between pathological response and improved outcomes is consistent with previous clinical studies (25, 33). Taken together, these findings should be interpreted with caution, and the prognostic value of pCR in this setting requires validation in larger, prospective trials with longer follow-up.

There is no unified standard for the number of neoadjuvant treatment cycles for resectable locally advanced Siewert type II AEG, leading to much controversy. Most Japanese studies adopt a two-cycle regimen, whereas studies such as FLOT4, MAGIC, and FFCD (16, 34, 35)opt for 3–4 cycles and consider it beneficial for patients. Our research results show that patients who completed 3–4 cycles of neoadjuvant therapy had a better pCR rate compared to those who completed 1–2 cycles of neoadjuvant therapy (p=0.015). However, there was no significant difference in the MPR rate and postoperative pathological downstaging rate. This finding may be due to the fact that two cycles of neoadjuvant therapy can achieve the goals of downstaging and downgrading, but if a higher pCR rate is desired, the number of neoadjuvant cycles needs to be increased. The selection of the number of cycles for neoadjuvant therapy in resectable locally advanced Siewert type II AEG should comprehensively consider various factors such as chemotherapy regimen, efficacy, adverse reactions, and drug efficacy. These factors all determine the choice of the number of nCI cycles, which in turn affects the patient’s treatment outcomes and clinical results.

Regarding the safety of preoperative treatment, the incidence of TRAEs in this study was 35.69%, with the most common hematological toxicity being leukopenia (15.84%),which less than that of neoadjuvant ECF/ECX and FLOT formula reported in the FLOT4 study (34). and the most common non-hematological toxicity being nausea and vomiting (16.83%). Most TRAEs were classified as grade I-II, and no treatment-related deaths occurred in this study. Compared with nCT related studies (16, 17, 27, 34), there was no additional risk of treatment. However,7 patients occurred irAEs, 3patients(2.97%) experienced grade 3–4 irAEs, including 1 case of Immune myocarditis (0.99%), 1 cases of Immune myositis (0.99%), and 1 case of Immunologically associated thyroid dysfunction (0.99%). This study highlights that myocarditis, though rare, can occur with a delayed onset (3 weeks after treatment initiation) and requires prompt recognition (via troponin/CK-MB monitoring) and multidisciplinary management (glucocorticoid therapy + cardiology collaboration). Consistent with guidelines from the American Society of Clinical Oncology (ASCO) for irAE management, early intervention led to favorable outcomes without long-term cardiac sequelae, providing practical experience for clinical practice.Severe irAEs, though rare (2.97%), require vigilant monitoring—particularly myocarditis, which, while occurring in only 1 patient here, carries life-threatening risks.Overall, these adverse events are considered safe and manageable, and no treatment-related deaths have occurred. ICIs treatment for AEG has resulted in significant therapeutic improvements, yet it is also accompanied by a series of unique adverse events. This necessitates that clinicians possess a high degree of vigilance and expertise, as meticulous monitoring and timely intervention. Most adverse events can be properly managed, thereby ensuring patient safety. In the future, with the deepening of research into the toxicity mechanisms related to ICIs, we can expect to develop more precise predictive tools and individualized treatment plans, to further optimize the application of ICIs in the treatment of AEG, and to enhance the overall treatment outcomes and quality of life for patients.

In our study, most patients (90.1%) underwent “Upper abdominal + Left Thoracotomy”, which is the prevailing practice at our center. Data from previous concurrent studies (36) at our center showed that the lymph node metastasis rate of Siewert type II AEG is highest in the upper perigastric + supra-pancreatic region (61.6%), followed by the lower perigastric + hepatoduodenal region (17.1%) and the lower mediastinum (5.4%), with no metastasis observed in the middle and upper mediastinum. This multi-regional lymph node metastasis pattern necessitates a surgical approach capable of comprehensively addressing both thoracic and abdominal lymph nodes, and the “Upper abdominal + Left Thoracotomy” approach precisely meets this requirement. Compared with the traditional Sweet procedure or the upper abdominal approach, this surgical method does not increase the incidence of postoperative complications. Nevertheless, a small proportion of patients in this study cohort underwent the upper abdominal approach, which was determined based on individualized selection according to the patients’ physical conditions and tumor characteristics.Currently, the optimal surgical approach for Siewert type II AEG remains debated.We emphasize that our findings reflect the outcomes of our institutional approach, and further prospective studies comparing surgical techniques (e.g., randomized trials of open vs. minimally invasive resection for Siewert type II AEG) are needed to define the optimal approach.

In addition, the incidence of surgical-related complications was 36.6%, with the most common postoperative complication being lung infection (28.7%), followed by deep vein thrombosis in the lower limbs (4.9%). The postoperative complications were relatively manageable, and no perioperative mortality events occurred. The average operative time was 266 minutes, with an average intraoperative blood loss of approximately 150ml, a median number of lymph node dissections of 28, and an average postoperative hospital stay of 9 days. There were no intraoperative complications, consistent with previous studies (27, 37) reporting on nCT or nCRT combined with surgical treatment. The results confirm the safety and feasibility of nCI in resectable locally advanced AEG patients, without increasing the difficulty and risk associated with surgery.

Our study has several inherent limitations. First, it was a single-center retrospective analysis without a contemporaneous nCT control group, which may introduce selection bias and limits the strength of causal inference. Second, the follow-up duration was relatively short, the survival endpoints remain immature, and the observed advantages of nCI should be regarded as preliminary; Longer follow-up is essential to fully evaluate its impact on long-term survival and recurrence. Third, biomarker analyses were limited. Routine testing for PD-L1 expression, MSI, TMB, and dMMR was not performed in all patients due to the retrospective nature of this study. Ongoing prospective studies at our center are expected to provide more comprehensive biomarker data and long-term clinical outcomes, thereby strengthening the confirmation of our preliminary results.

## Conclusion

Neoadjuvant chemotherapy combined with immunotherapy show a promising efficacy in the treatment of resectable locally advanced Siewert type II AEG, with high rates of pCR and MPR, as well as good tolerance and safety. Moreover,3–4 cycles of nCI may provide better therapeutic efficacy than 1–2 cycles. Additionally, this treatment regimen has potential long-term benefits in reducing recurrence and metastasis rates and prolonging survival.Validation in larger prospective studies with extended follow-up is required to determine the long-term survival impact of this regimen.

## Data Availability

The original contributions presented in the study are included in the article/supplementary material. Further inquiries can be directed to the corresponding author.
